# The nuclear-cytoplasmic trafficking of a chromatin-modifying and remodelling protein (KMT2C), in osteosarcoma

**DOI:** 10.18632/oncotarget.25755

**Published:** 2018-07-17

**Authors:** Caterina Chiappetta, Chiara Puggioni, Raffaella Carletti, Vincenzo Petrozza, Carlo Della Rocca, Claudio Di Crisfofano

**Affiliations:** ^1^UOC of Pathology, Department of Medical-Surgical Sciences and Bio-Technologies, Sapienza University of Rome, Latina, Italy

**Keywords:** osteosarcoma, KMT2C, tumorigenesis, metastasis, nuclear-cytoplasmic trafficking

## Abstract

Osteosarcoma is the most common paediatric primary non-hematopoietic bone tumor; the survival is related to the response to chemotherapy and development of metastases. KMT2C is a chromatin-modifying and remodelling protein and its expression has never been studied in osteosarcoma. The aim of this study was to understand the role of KMT2C in the osteosarcoma carcinogenesis and metastatic progression to identify a new molecular target and to provide new therapeutic approach.

We performed the immunohistochemical and gene expression analysis of KMT2C in 32 samples of patients with diagnosis of osteosarcoma with known clinic-pathological data and we analysed the expression of genes involved in the metastatic pathway in four osteosarcoma cell lines by blocking the *KMT2C* expression using siRNA.

We found a nuclear-cytoplamic trafficking of KMT2C and the cytoplasmic localization was higher than the nuclear localization (*p* < 0.0001). Moreover, the percentage of cells with cytoplasmic positivity increased from low grade primary tissue to metastatic tissues. The cytoplasmic localization of KMT2C could lead to a change in its function supporting osteosarcoma carcinogenesis and progression. Our hypothesis is that KMT2C could affect the enhancer activity of genes influencing the invasive properties and metastatic potential of osteosarcoma.

## INTRODUCTION

Despite osteosarcoma being considered an uncommon cancer, it represents the primitive, non-haematopoietic, malignant tumor of the bone that is commonly found [1–2]. Osteosarcoma arises mainly in the long bones of the extremities and the main feature is the detection of osteoid matrix produced by neoplastic cells [3]. The therapeutic protocols include neoadjuvant conventional chemotherapy, surgical resection of the primary tumor and post-operative chemotherapy [4]. Patients’ survival is related to the development of metastasis and the response to chemotherapy [5]. The 5-year survival rate for patients with osteosarcoma without evidence of metastasis is 60–65%, whereas it is only 20–28% for osteosarcoma patients with metastases at the time of diagnosis [6–7]. <90% of tumor necrosis evaluated on surgical specimen after neoadjuvant chemotherapy is considered a poor prognostic indicator (“non-responder” patients).

To date, clinical-pathological parameters and molecular markers that could be used as predictors of response to chemotherapy or to be able to identify patients with elevated risk of developing metastasis, have not yet been identified. Moreover, the molecular events causing development, progression and metastatic process of osteosarcoma are still unknown [4, 8]. The somatic genome of the osteosarcoma is considered complex and characterized by tumor heterogeneity; indeed, increased number of mutations, structural and copy number variations and genomic instability are typical features of osteosarcoma. Moreover, the mutation rate of osteosarcoma is the highest among all pediatric tumors [9–10].

Therefore, the identification of genetic variations involved in osteosarcoma carcinogenesis and progression is a key point to understand the mechanisms that would allow the creation of personalized protocols, which could significantly increase treatment effectiveness and improve the outcomes. In our previous study of Whole Exome Sequencing (WES) analysis [11], we confirmed that the osteosarcoma karyotype is complex; indeed, we did not find a genetic variation common to all high-grade osteosarcoma diagnostic biopsies analyzed, but we found that the *KMT2C* gene, a key component of histone H3 lysine 4 methyltransferase complexes [12], showed the highest number of variations in most of the samples being analyzed. It has been shown that highly conserved epigenetic regulators are frequently mutated in cancer and some studies suggest that variations in coding sequences of regulating elements, which act on enhancers to recognize specific transcriptional factors, may be the cause of tumor development [13]. Particularly, KMT2C acts together with hormone receptors and transcription factors involved in pathways that promote cell growth and performs its action in enhancer regions [12]. KMT2C is mutated in a wide spectrum of neoplasms, it has been linked to tumorigenesis [12], it has never been studied in osteosarcoma and its role in metastatic progression is unknown. KMT2C is implicated in the regulation of the *TP53* gene [14], which encodes a tumor suppressor protein frequently altered in osteosarcoma [8]. Moreover, it has been shown that *KMT2C* mutations may alter its co-operation with the estrogen receptor [15] and this hormone plays a key role in bone development, remodeling and during matrix production [16]. Our earlier data showed that KMT2C could be involved in osteosarcoma carcinogenesis [11]. Therefore, the aim of this study was to confirm the role of KMT2C in the osteosarcoma carcinogenesis on a large cohort of osteosarcoma samples and evaluate it in osteosarcoma metastatic progression on osteosarcoma cell lines. The goal was to identify a molecular target to provide the basis for a new therapeutic approach and improve the survival of patients with osteosarcoma.

## RESULTS

### Immunohistochemical results

**Figure 1:**
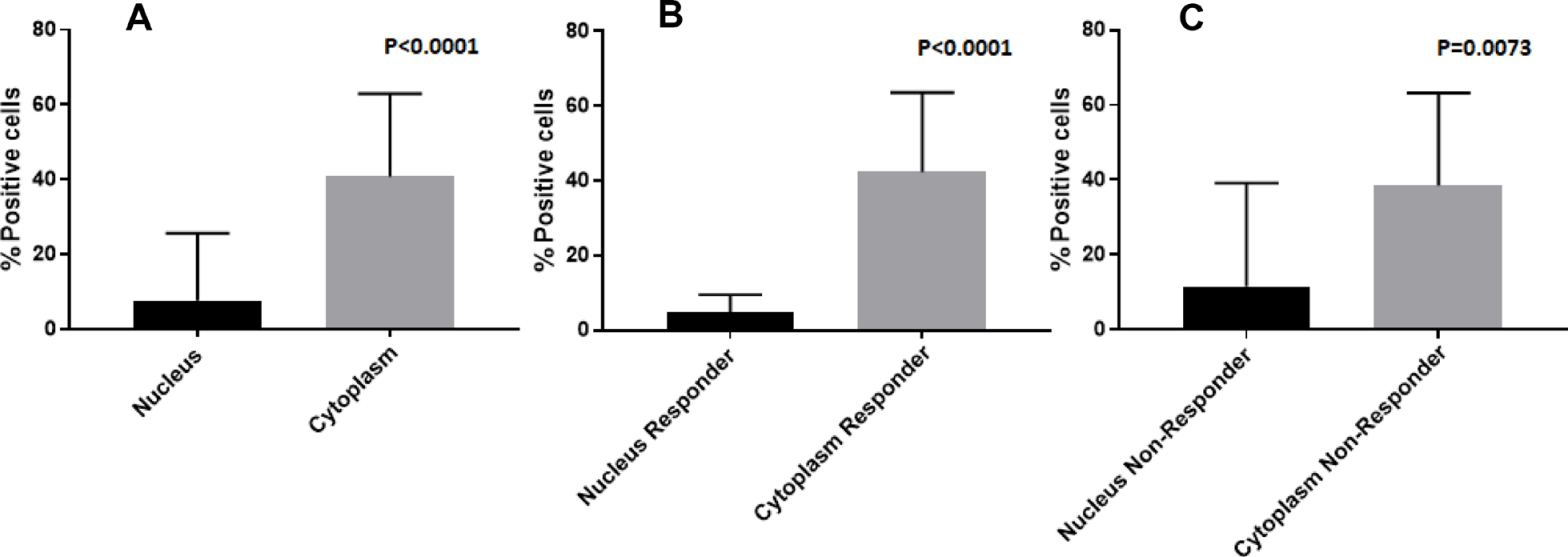
Histogram of the average percentage of cells that show nuclear and cytoplasmic positivity for KMT2C by immunohistochemical staining; (A) overall; (B) responder; (C) non-responder

**Figure 2:**
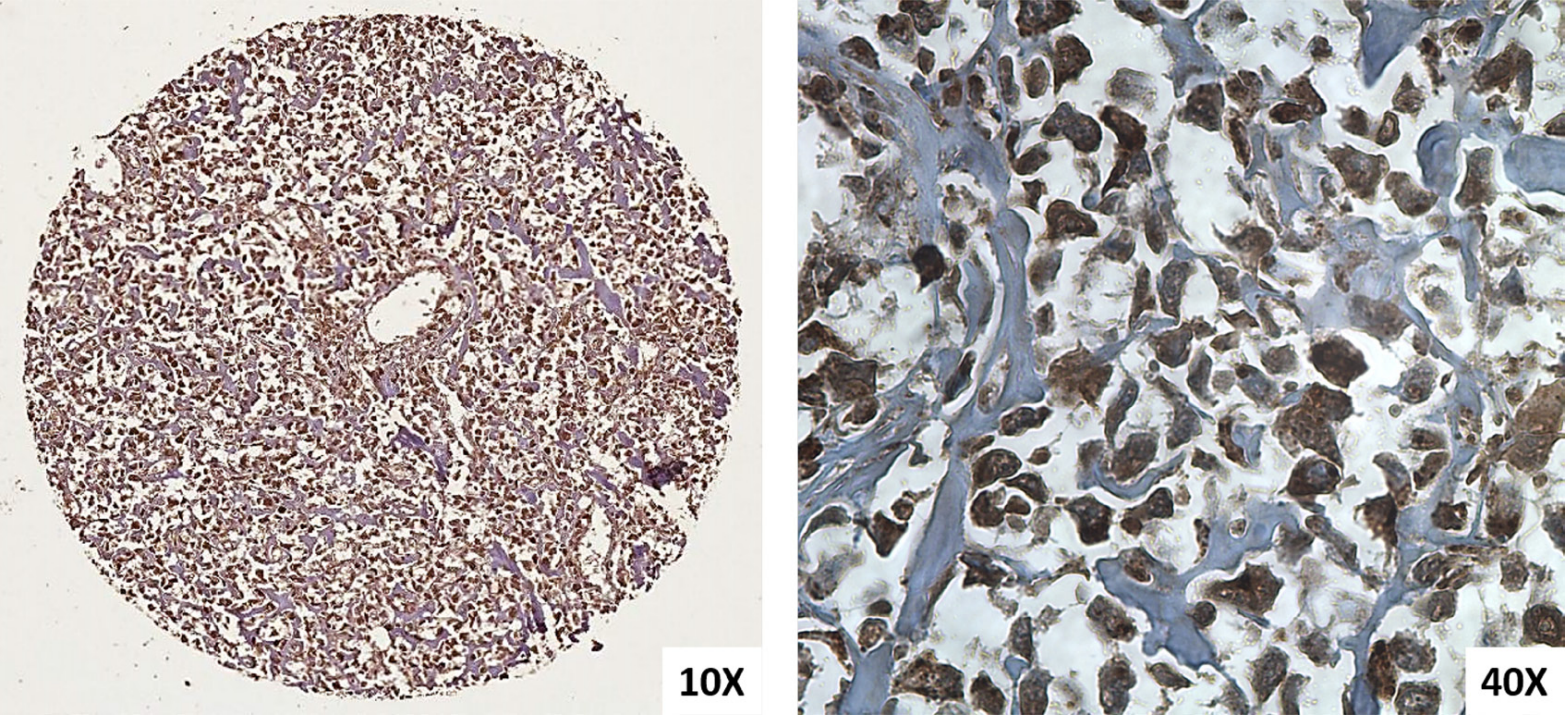
Immunohistochemical KMT2C expressions in osteosarcoma performed on TMA spot (10× and 40×)

We evaluated the KMT2C immunohistochemical expression of 32 samples on Tissue MicroArray (TMA) sections and we observed that all osteosarcoma samples showed cytoplasmic expression of KMT2C and 31/32 (97%) osteosarcoma samples also showed nuclear localization (Figures 1, 2).

The mean value of nuclear and cytoplasmic protein localization was 8.9 (range 0–80) and 38.6 (range 3–88) respectively. Only one sample did not show the nuclear localization of KMT2C; this was a patient with high-grade osteosarcoma of the femur (stage IIB), follow-up was 6 years, he responded to neoadjuvant chemotherapy and did not develop metastasis.

The average percentage of nuclear and cytoplasmic positive cells is described in Table 1.

**Table 1:** Results of KMT2C immunohistochemical analysis and *KMT2C* gene expression analysis

	*N*	Nuclear positive cells (%)	Cytoplasmic positive cells (%)	Relative mRNA expression	
**Overall**	**32**	8.9^*^	38.6^*^		^*^p < 0.0001
High grade osteosarcoma (NOS)	23	11.1	38.9	5.4	
Parosteal osteosarcoma	3	3.7	33.0	3.2	
Central low-grade osteosarcoma	3	4.0	26.3	2.2	
Telangiectatic osteosarcoma	2	3.0	66.5	3.6	
Periosteal osteosarcoma	1	1.0	30.0	1.1	
Responder	13	4.9^*^	39.8^*^	4.7	^*^p < 0.0001
Non-Responder	11	12.9^*^	39.4^*^	5.4	^*^p = 0.0073
G1–G2	9	4.3	26.6	2.2	
G3–G4	23	10.5	42.6	5.5	
Overall metastasis	3	7.0	54.0	5.8	
Pulmonary metastasis	1	15.0	68.0	2.0	
Bone metastasis	2	3.0	47.0	7.7	
Relapse	4	2.5	26.2	2.7	

The IHC values are expressed as percentage of tumor cells with nuclear and cytoplasmic positivity.

The normal bone tissue controls mainly showed a nuclear expression of KMT2C and occasionally a cytoplasmic protein localization.

### *KMT2C* mRNA expression

We observed that *KMT2C* was up-regulated and down-regulated in 84.4% (27/32) and 15.6% (5/32) of osteosarcoma samples respectively compared to osteoporosis tissue sample used as control. The results of *KMT2C* gene expression analysis are described in Table 1. We did not find a statistical association between *KMT2C* gene expression and clinical-pathologic features of patients or the cellular localization of the protein.

### Immunofluorescence protein localization in cell culture

**Figure 3:**
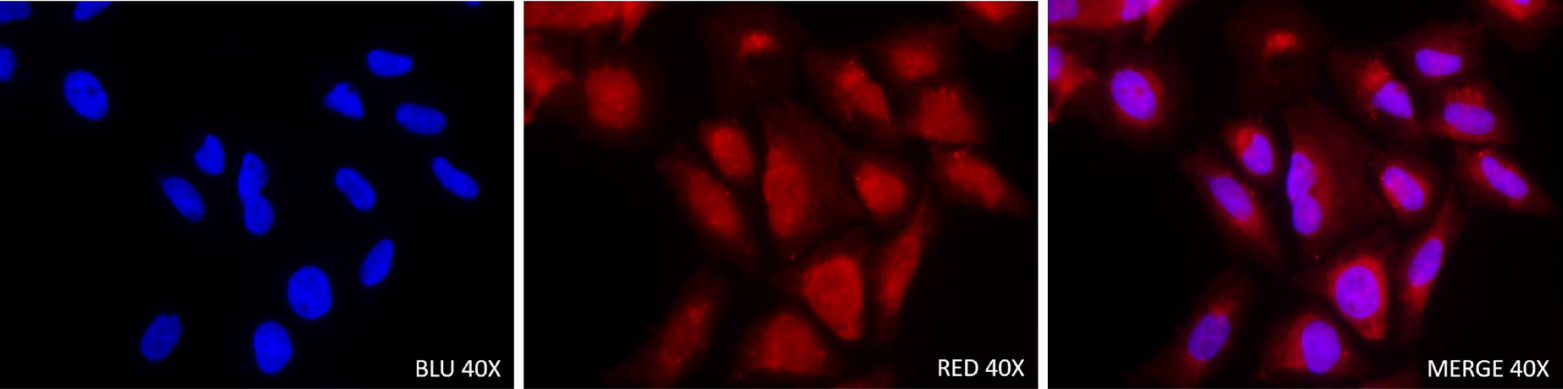
Immunofluorescence staining with KMT2C antibody in Saos-2 osteosarcoma cell line (40×)

The immunofluorescence was performed on confluent osteosarcoma culture cells and we found that KMT2C localized at both nuclear and cytoplasmic levels in all primary and metastatic cell lines analysed (Figure 3).

### Effective targeting of KMT2C with RNA interference

**Figure 4:**
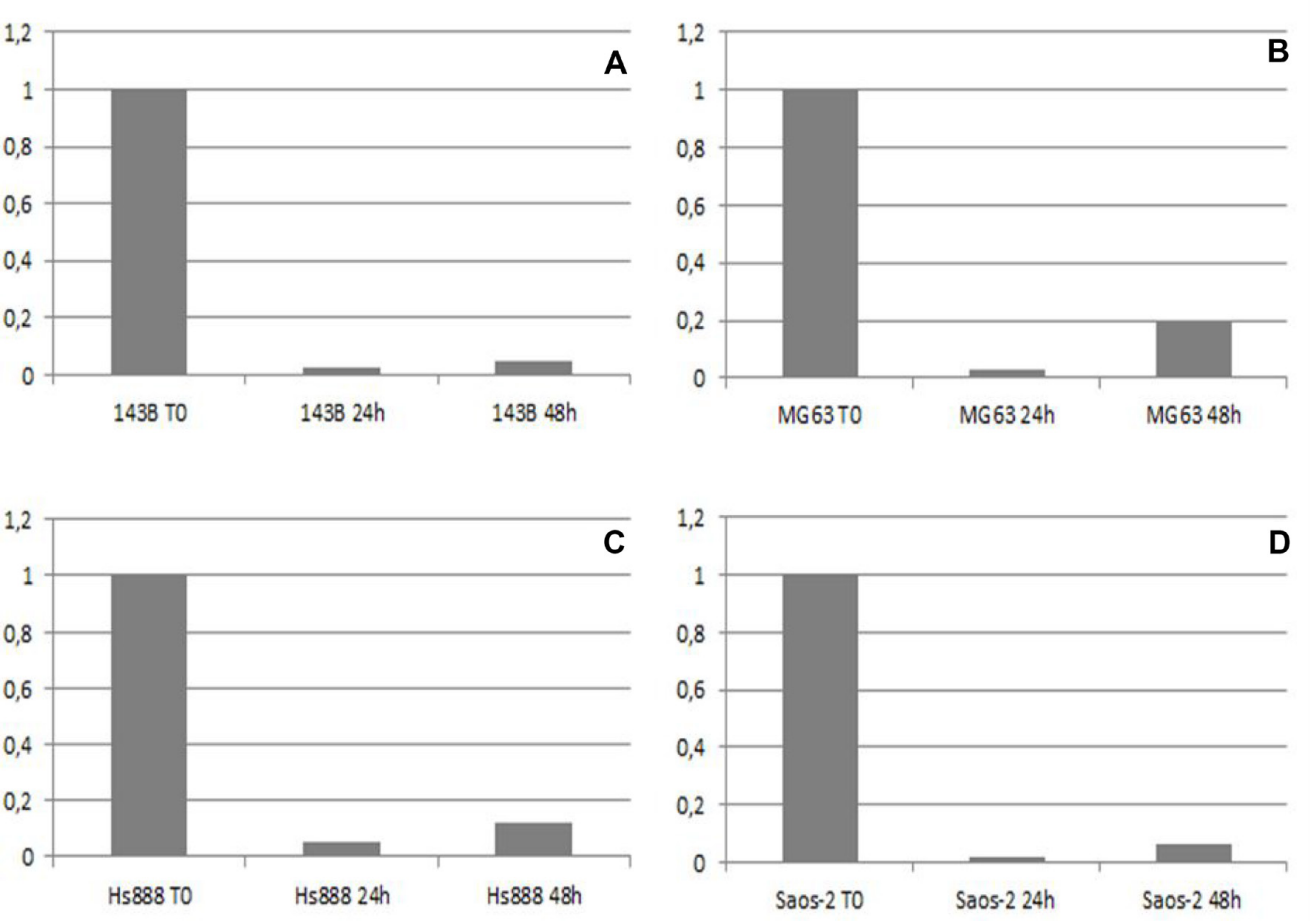
The mRNA expression level of KMT2C detected by real-time PCR before (T0), 24 and 48 hours after KMT2C siRNA transfection in 143B (A), MG63 (B), Hs888 (C) and Saos-2 (D) osteosarcoma cell lines

A duration of 24 and 48 hours after transfection with *KMT2C* siRNA, quantitative real-time PCR assay was performed to analyse the expression of *KMT2C* mRNA. The expression of *KMT2C* mRNA was suppressed in the groups transfected with *KMT2C* siRNA compared with the group without transfection (T0, Figure 4). All primary osteosarcoma cell lines showed an expression decrease of 97% (Figure 4A–4D) whereas the metastatic cell line Hs888 showed an expression decrease of 94% (Figure 4C). The expression of β-actin mRNA showed no change as an internal control.

### Expression modulation of genes involved in the metastatic pathway after KMT2C silencing

Using Applied Biosystems™ Analysis Software, we analyzed 92 genes involved in metastatic pathway and 4 housekeeping genes using mRNA of osteosarcoma cell lines before and after 24 h *KMT2C* siRNA transfection. We found that of 4 housekeeping genes tested, *HPRT1* appeared as the most reliable control genes for the analysis of RealTime PCR data.

We noted that 3 genes (3/92, 3.3%, *CXCL12*, *SERPINE1* and *HTATIP2*) were upregulated only in metastatic cell line Hs888.

After *KMT2C* siRNA transfection we observed that 8 genes (8/92, 8.7%, *FN1*, *CD44*, *MMP2, TIMP1, CTNNA1, CXCL12*, *SERPINE1* and *HTATIP2*) showed different expression levels compared to the previously performed analysis (Table 2).

**Table 2:** Gene expression values of the 8 genes that showed different expression levels before and after (bold) mRNA *KMT2C* silencing in Osteosarcoma (OS) cell lines

GENE	OS primary cell lines			OS metastatic cell line
	**143B**	**MG63**	**Saos-2**	**Hs888**
FN1	23.6	3.0	3.2	71.8
**FN1**	**30.6**	**3.41**	**1.9**	**64.1**
CD44	640.9	21.9	0.3	23.7
**CD44**	**5.8**	**16.6**	**0.6**	**36.1**
MMP2	1	11.5	4.0	19.5
**MMP2**	**1.9**	**20.5**	**6.9**	**24.8**
TIMP1	5.0	1.3	1.9	14.0
**TIMP1**	**3.7**	**1.0**	**1.1**	**14.0**
CTNNA1	0.7	1.8	0.9	3.1
**CTNNA1**	**0.8**	**2.6**	**0.7**	**2.2**
CXCL12	0.0	0.0	0.0	11.3
**CXCL12**	**0.0**	**0.0**	**0.0**	**6.6**
SERPINE1	0.0	0.0	0.0	15.0
**SERPINE1**	**0.0**	**0.0**	**0.0**	**9.4**
HTATIP2	0.0	0.0	0.0	1.4
**HTATIP2**	**0.0**	**0.0**	**0.0**	**3.1**

We found that there was an upregulation of both *CD44* and *FN1* (except in the Saos-2 cell line) and following *KMT2C* mRNA silencing, cell lines 143B and MG63 showed an increase in *FN1* expression levels and a decrease in *CD44* levels while the Saos-2 and the Hs888 metastatic line behaved in the opposite way. After *KMT2C* mRNA silencing, we observed an increase in expression levels of *MMP2* in all four cell lines while *TIMP1* levels decreased in all primary cell lines while it remained constant in metastatic cell line Hs888. We observed that MG63 cell line and Hs888 metastatic cell line showed an upregulation of gene codifying α-CATENIN (*CTNNA1*); following *KMT2C* mRNA silencing, *CTNNA1* gene showed a lower gene expression value in the metastatic line and a higher value in the MG63 cell line.

Moreover, because of the silencing, we found that *SERPINE-1*, *CXCL12* and *HTATIP2* genes showed a modulation of gene expression values compared to before *KMT2C* mRNA silencing only in the Hs888 metastatic line; we found that the expression values of *SERPINE*-1 and *CXCL12* genes was decrease while we found an increase in the expression value of the *HTATIP2* gene (Table 2).

Finally, the other 84 genes (84/92, 91.3%) did not show variations in expression levels after *KMT2C* mRNA silencing in all osteosarcoma cell lines.

## DISCUSSION

KMT2C belongs to the family of histone-modifying proteins KMT2, it catalyses the monomethylation of H3K4 [17] and recent studies provided significant data that bind dysregulation of genes coding chromatin-modifying and remodeling proteins to cancer [13]. The monomethylation of H3K4 occurs more frequently on enhancer regions [18]; so, it is possible that mutations in proteins like KMT2C could inactivate tumor suppressors or activate oncogenes [13]. Moreover, chromatin modifiers are already recognized as a cancer therapy target and clinical trials for the treatment of MLL1/KMT2A-fusion leukemia provide evidence for the development of drugs targeting the members of KMT2 family [19].

We found that most of analysed osteosarcoma samples (97%) showed both nuclear and cytoplasmic immunohistochemical expression of KMT2C and the percentage of cytoplasmic positive cells was higher than the percentage of nuclear positive cells. Moreover, the cellular localization of KMT2C, both nuclear and cytoplasmic, was confirmed by immunofluorescence analysis. The nuclear localization of KMT2C agrees with its function as co-activator of transcriptional factors while the cytoplasmic localization, that was already described for KMT2C [20], showed that there was an altered intracellular localization of KMT2C (nuclear-cytoplamic trafficking). Recent evidence shows that specific variations of *KMT2C* can lead to different effects, including the inactivation of the protein or a change in the enzymatic activity [21]. The cytoplasmic localization of KMT2C could likely lead to a loss, gain, or change in its function supporting osteosarcoma tumorigenesis. Moreover, the only one sample that did not show the nuclear localization of the protein showed the highest value of *KMT2C* mRNA expression. Since this is an isolated case, we could speculate that the protein not present at the nuclear level cannot perform its canonical function, and this leads to an increase of the transcription of the *KMT2C* gene.

Even if we did not find a statistically significant difference between primary and metastatic tumors, we observed that between low grade primary tumor and high grade primary tumor there was an increase of the percentage of cells with nuclear positivity while this value decreased in metastatic tissues. On the contrary, the *KMT2C* gene expression value as well as the percentage of cells with cytoplasmic positivity increased from low grade primary tissue to metastatic tissues. This observation could corroborate our hypothesis that the cytoplasmic localization of KMT2C probably determined a change in its function even during metastatic progression.

After *KMT2C* mRNA silencing, we observed that *CD44* and *FN1* underwent a modulation of their values. CD44, a trans-membrane glycoprotein, communicates with the cytoskeleton through its cytoplasmic domain [22]. The association between CD44 expression and tumor biology of osteosarcoma is known [23]. *FN1* encodes fibronectin, which may be present in dimeric or multimeric form at the cell surface and in the Extracellular Matrix (ECM) [24]. It is involved in cell adhesion, migration and metastasis and association between fibronectin expression and invasiveness has been documented in several tumors [12]. Our data highlighted that *KMT2C* modulated the gene expression of *CD44* and *FN1* probably influencing the attachment of osteosarcoma cells to ECM, a necessary step for osteosarcoma cells to metastasize [25].

Coherently with other studies carried out on osteosarcoma cell lines, we observed that *MMP2* was upregulated in all osteosarcoma cell lines [26] especially in metastatic cell line. MMPs are proteolytic enzymes that tightly control the degradation of ECM [27] and their altered expression was correlated with poor prognosis in different human cancers [28], including osteosarcomas [26]. Regulation of MMPs is linked to tissue inhibitor of MMPs (TIMP) and their action determines the inhibition of cell invasion [28]. According to a recent study [29], we observed a negative correlation between *MMP2* and *TIMP1* and after *KMT2C* mRNA silencing we found an increase of *MMP2* gene expression value in all cell lines and a decrease of *TIMP1* expression values in primary osteosarcoma cell lines while in metastatic cell line remained constant. Therefore, our study shows that *KMT2C* could be involved in ECM modifications that lead to metastatic progression and it could regulate the MMP2 activity in osteosarcoma progression.

Surprisingly, we observed that two cell lines, particularly the metastatic cell line Hs888, showed an upregulation of gene codifying α-CATENIN (*CTNNA1*) and after *KMT2C* mRNA silencing the gene expression value of *CTNNA1* decreased only in metastatic cell line. α-CATENIN is recognized as a putative tumor suppressor both in maintenance of cell-cell adhesion and regulating β-CATENIN activity in the Wnt/β-catenin pathway [30], also involved in osteosarcoma carcinogenesis [31]. Our data highlighted that α-*CATENIN* is expressed in osteosarcoma cell lines and we could argue that α-*CATENIN* expression values could decrease only during metastasis development in osteosarcoma and that this process could be regulated by *KMT2C*.

From gene expression analysis we found that three genes (*CXCL12, SERPINE1, HTATIP2*) were upregulated only in metastatic cell line and that these values were modulated after *KMT2C* mRNA silencing. Particularly, we found that the *CXCL12* gene expression decreased after *KMT2C* mRNA silencing. Chemokine 12 is a chemoattractive cytokine involved in the metastasis process in some cancer, and the binding to its receptor leads to the activation of several downstream pathways that regulate survival, proliferation and migration [32]. Recently, the chemokine CXCL12/CXCR4 signaling has been extensively investigated in osteosarcoma due to the relevance in metastasis progression and poor patient outcome [33]. In addition, it has been showed that the CXCL12-CXCR4 axis activation leads to increase of metalloproteinase expression, as *MMP2*, causing degradation of ECM at the invasion front [34]. Coherent with this data we found that *MMP2* gene expression level was higher in metastatic cell line than the other osteosarcoma cell lines. So, as in other types of tumors [33], the use of small molecule antagonists of this chemokine receptors may be useful to interfere with tumor progression and metastasis in osteosarcoma patients.

Glycoprotein SERPINE1 (PAI-1) has been shown to be involved in promoting cell adhesion and a higher expression of *SERPINE1* in tumor cells contributes to cancer progression including metastasis [35]. In human osteosarcoma cells its expression is correlated with an increased risk of lung metastasis [36]. In line with literature, through our study we found that this gene was upregulated only in osteosarcoma metastatic cell line and after *KMT2C* mRNA silencing, we observed a decrease of gene expression value. Given that the mechanism by which SERPINE1 promotes the invasion and metastasis of osteosarcoma cells remains unknown [36], following our analysis, we hypothesize a role of *KMT2C* in this process. Therefore, it would be interesting to deepen the knowledge of the role of SERPINE1 in osteosarcoma progression to obtain a marker for the prediction of metastasis development. Finally, we observed that gene *HTATIP2*, which codifies a metastasis suppressive protein, was upregulated only in metastatic cell line and after *KMT2C* mRNA silencing we observed an increase of the gene expression value. HTATIP2 is involved in the control of cell apoptosis, growth, metastasis, angiogenesis and DNA repair; it has been found to be expressed in different tumors [37–38], but it has never been described in osteosarcoma. For these reasons, it would be interesting to study the possible involvement of this protein in osteosarcoma progression.

In conclusion, we obtained valuable information about KMT2C in osteosarcoma: we found a nuclear-cytoplasmic trafficking that could have a role in osteosarcoma carcinogenesis and progression probably linked to the consequent change of its function. Our hypothesis is that KMT2C affecting negatively or positively the enhancer activity of genes involved in osteosarcoma progression, it could play a role of oncogene or oncosuppressor, particularly in the degradation and attachment of tumor cells to ECM. However, further studies are needed to better understand the role of KMT2C in osteosarcoma carcinogenesis and progression because epigenetic therapy is promising for cancer treatment in the light of recent developments in this field. Therefore, this study could offer the opportunity to identify a potential therapeutic target to obtain personalized therapy and increase the survival rate of young patients affected by osteosarcoma.

## MATERIALS AND METHODS

### Population of study

The population of this study included 32 patients with osteosarcoma from the files of the Department of Medical and Surgical Sciences and Biotechnologies, Sapienza University of Rome, Latina, Italy. The patients, informed during the follow-up, gave their consent for the study. The WHO 2013 classification of bone tumors was used to classify the bone lesions. The clinical-pathological features are described in Table 3. Patients had a mean follow-up time of 9.5 years (range 1–18 years). Out of these 24 patients who received neoadiuvant chemotherapy, 13 (identified as “responder”, 54%) didn’t develop metastasis, while 11 patients (identified as “non-responder”, 46%) did not respond to neoadjuvant chemotherapy, developed metastasis and died. Two patients had histologically proven bone metastases at diagnosis and for one patient we had the tissue of pulmonary metastasis. Moreover, for 4 patients we collected relapse tissue. There were no instances of inherited cancer predisposition syndromes associated with osteosarcoma among our samples. A bone tissue sample of patient with diagnosis of osteoporosis was used as control for gene expression analysis. All the tissue samples were reviewed on microscopy examinations by two dedicated pathologists.

**Table 3:** Clinical-pathological features of osteosarcoma samples

**Sex**		
13/32	40.6%	Male
19/32	59.4%	Female
**Age**		
9/32	28%	≤18 years
23/32	72%	≥18 years
**Type of osteosarcoma**		
23/32	72%	High grade osteosarcoma (NOS)
3/32	9%	Parosteal osteosarcoma
3/32	9%	Central low-grade osteosarcoma
2/32	6%	Telangiectatic osteosarcoma
1/32	3%	Periosteal osteosarcoma
^*^Grade		
9/32	28%	G1–G2
23/32	72%	G3–G4
**Site of osteosarcoma**		
26/32	81%	Upper limbs
2/32	6%	Lower limbs
3/32	9%	Jaw/Mandible
1/32	3%	Lumbar vertebra
**Stage**		
4/32	12.5%	IB
5/32	15.5%	IIA
21/32	66%	IIB
1/32	3%	IVA
1/32	3%	IVB
**Relapse**		
4/32	12.5%	

* Sec. AJCC System.

### Immunohistochemical analysis of KMT2C

TMA sections (2 μm), of osteosarcoma samples, already used for other our studies [39], were deparaffinized and rehydrated in graded ethanol. Endogenous peroxidase activity was blocked by 3% hydrogen peroxide for 10 minutes. Antigen retrieval was performed in EDTA (pH 8.0) for 15 minutes. Blocking not specific sites was performed with Protein Block Serum-Free (DAKO, Carpinteria, CA, USA). The tissue sections were then incubated with goat IgG polyclonal antibody anti-MLL3 (Q-20, Santa Cruz Biotechnology, Dallas, TX, USA) 1:50, at 4° C overnight. After incubation, specimens were washed with TBS buffer and with the secondary biotinylated antibody and subsequently with the streptavidin–biotin–peroxidase (DAKO LSAB Kit peroxidase; DAKO, Carpinteria, CA, USA). The samples were then washed with TBS buffer and incubated with freshly prepared DAB + substrate–chromogen buffer at room temperature. After gently rinsing with ddH_2_O, slides were counterstained with hematoxylin and mounted with permanent mounting media. Both positive and negative internal and external controls were used in each experiment. The immunostaining was evaluated by two experienced blinded pathologists using a double score based on staining localization (cytoplasmic/nuclear) and on the percentage of positive neoplastic cells. The percentage of positive neoplastic cells for each sample was calculated as the average of the positive neoplastic cells of the three TMA spot.

### GENE expression analysis – RealTime PCR

Total RNA from Formalin-Fixed Paraffin Embedded (FFPE) samples was extracted using RecoverAll™ Total Nucleic Acid Isolation Kit for FFPE (ThermoFisher Scientific, Waltham, MA, USA) according to the manufacturer’s instructions. We confirmed the purity and quantity of RNA by NanoDrop ND-1000 Spectrophotometer (ThermoFisher Scientific, Waltham, MA, USA). The RNA was reverse transcribed into cDNA with High-Capacity cDNA Reverse Transcription Kit (Thermo Fisher Scientific, Waltham, MA, USA). PCR product of human *KMT2C* (Gene ID: 58508, Hs01005521_m1, ThermoFisher Scientific, Waltham, MA, USA) was detected using gene-specific primer and probe labeled with reporter dye FAM, which yielded a predicted amplicon of 58 bp. β-ACTIN (Gene ID: 60, Hs01060665_g1, ThermoFisher Scientific, Waltham, MA, USA) was used as an internal standard, which yielded a predicted amplicon of 171 bp. TaqMan real-time quantitative PCR for *KMT2C* mRNA was performed on an ABI PRISM 7500 Fast Real-Time PCR System (Applied Biosystem, Foster City, CA, USA). PCR reaction was carried out in triplicate on 96-well plate with 10 uL per well using 1X TaqMan Master Mix. After an incubation for 2 minutes at 50° C and 10 minutes at 95° C, the reactions continue for 40 cycles at 95° C for 15 seconds and 60° C for 1 minute. At the end of the reaction, the results were evaluated using the ABI PRISM 7500 software (Applied Biosystem, Foster City, CA, USA). The cycle threshold (Ct) values for each set of 3 reactions were averaged for all subsequent calculations. The 2^−ΔΔCt^ method was used to calculate relative changes in gene expression.

### Cell culture

143B, MG63, Saos-2, and Hs888 human osteosarcoma cells lines obtained from American Type Culture Collection (ATCC, Manassas, VT, USA) were cultured in Dulbecco’s modified Eagle’s medium supplemented with 10% to 15% fetal bovine serum, 1mM sodium piruvate, 100 U/mL of penicillin, and 100 mg/mL of streptomycin, at 37° C and 5% CO_2_, according to ATCC recommendations. 143B, MG63 and Saos-2 were osteosarcoma primary cell lines while Hs888 was an osteosarcoma metastatic cell line (lung). All cell lines derived from young adults with osteosarcoma diagnosis, two males (MG63 and HS888) and two females (143B and Saos-2).

### Immunofluorescence analysis of KMT2C

For immunofluorescent assay, the 143B, MG63, Saos-2 and Hs888 cells were fixed in a 4% paraformaldehyde solution for 5 minutes at 4° C and then cells were permeabilized with PBS + 0.01% Tween 20 for 20 minutes at room temperature. Subsequently, the cells were incubated with goat IgG polyclonal antibody anti-MLL3 (Q-20, Santa Cruz Biotechnology, Dallas, TX, USA) 1:50, at 4° C overnight. The following day, cells were washed with PBS buffer + 0.01% Tween 20 and subsequently cells were incubated with secondary antibody (Alexa Fluor 594 donkey anti-goat IgG; Invitrogen, Carlsbad, CA, USA) for 1 hour at room temperature. Then, the nuclei were counterstained with DAPI (Invitrogen, Carlsbad, CA, USA) for 10 minutes. Negative control was obtained by omitting the primary antibody. The fluorescence signal was examined with a fluorescence microscope Olympus IX50 with appropriate filters. Under 40X magnification, images of positive representative fields were captured.

### KMT2C siRNA transfection

siRNA sequences targeting *KMT2C* and a negative control siRNA were designed and synthesized by Invitrogen (Life Technologies, Foster City, CA, USA). The siRNAs were designed according to the *KMT2C* complementary DNA (cDNA) sequence (Gene ID: 58508). 143B, MG63, Saos-2, and Hs888 cells were seeded without antibiotics at 1/10^5^ per well in 6-well plates 2 days before transfection. When cells were 90% confluent, the cells were washed with PBS and siRNAs were transfected into 143B, MG63, Saos-2 and Hs888 cells using Lipofectamine RNAiMAX (Life Technologies, Foster City, CA, USA), according to the manufacturer’s instructions. The cells were harvested before and 24 and 48 hours after transfection with *KMT2C* siRNA; then the cells were subjected to RealTime PCR to determine the mRNA expression of *KMT2C*. Total RNA was extracted with a SV Total RNA Isolation System (Promega, Madison, WI, USA), according to the manufacturer’s instructions. We confirmed the purity and quantity of RNA by NanoDrop ND-1000 Spectrophotometer (Thermo Fisher Scientific, Waltham, MA, USA). The RNA was reverse transcribed into cDNA with High-Capacity cDNA Reverse Transcription Kit (Thermo Fisher Scientific, Waltham, MA, USA). PCR products for human *KMT2C* and β-ACTIN, used as an internal standard, were detected using gene-specific primers and probes labeled with reporter dye FAM, which yielded a predicted amplicon of 58 and 171 bp, respectively. TaqMan RealTime quantitative PCR for *KMT2C* and β-ACTIN mRNA was performed on an ABI PRISM 7500 Fast Real-Time PCR System (Applied Biosystem, Foster City, CA, USA). PCR reaction was carried out in triplicate on 96-well plate with 10 uL per well using 1× TaqMan Master Mix. After an incubation for 2 minutes at 50° C and 10 minutes at 95° C, the reactions continue for 40 cycles at 95° C for 15 seconds and 60° C for 1 minute. At the end of the reaction, the results were evaluated using the ABI PRISM 7500 software (Applied Biosystem, Foster City, CA, USA). The Ct values for each set of 3 reactions were averaged for all subsequent calculations. The 2^-ΔΔCt^ method was used to calculate relative changes in gene expression.

### Taqman array human tumor metastasis

Total RNA was extracted from harvested cells with a SV Total RNA Isolation System (Promega, Madison, WI, USA), according to the manufacturer’s instructions. We confirmed the purity and quantity of RNA by NanoDrop ND-1000 Spectrophotometer (Thermo Fisher Scientific Inc., Waltham, MA, USA).

The RNA was reverse transcribed into cDNA with highcapacity cDNA Reverse Transcription Kit (Thermo Fisher Scientific, Waltham, MA, USA). The cells were subjected to RealTime PCR to determine the mRNA expression of 92 gene involved in the metastatic pathway using TaqMan^®^ Array 96-well Human Tumor Metastasis Plate (Applied Biosystem, Foster City, CA, USA) using 10 uL per well using 5 uL of TaqMan Advanced Master Mix. Four housekeeping genes, *GAPDH*, *HPRT1*, *GUSB* and *18S*, were used as internal controls. After UNG (Uracil N-Glycosylase) incubation of 2 minutes at 50° C and 20 seconds of polymerase activation at 95° C, the reaction continues for 40 cycles at 95° C for 3 seconds and 60° C for 30 seconds. At the end of the reaction, the results were evaluated using the ABI PRISM 7500 software (Applied Biosystem, Foster City, CA, USA). The experiment was repeated in triplicate for each osteosarcoma cell line and the Ct values for each set of three reactions were averaged for all subsequent calculations. The 2^−ΔΔCt^ method was used to calculate relative changes in gene expression.

### Statistical analysis

Statistical analysis was performed using SPSS software (SPSS, Chicago, IL, USA). Comparison between parameters was calculated by paired and unpaired *t*-test. Correlations between variables were assessed by simple linear regression analysis. *P* value < 0.05 was considered statistically significant.

## Abbreviations

KMT2C/MLL3: lysine (K)-specific MethylTransferase 2C
siRNA: silencer RNA
WES: Whole Exome Sequencing
H3: Histone 3
TP5: Tumor Protein 53
TMA: Tissue MicroArray
HPRT1: Hypoxanthine PhosphoRibosylTransferase 1
PCR: Polymerase Chain Reaction
CXCL12: C-X-C motif chemokine Ligand 12
SERPINE1/PAI1: Eerpin family E member 1
HTATIP2: HIV-1 Tat Interactive Protein 2
FN1: Fibronectin 1
MMP2: Matrix Metallopeptidase 2
TIMP1: TIMP metallopeptidase inhibitor 1
CTNNA1: Catenin Alpha 1
H3K4: Histone 3 Lysine 4
MLL1/KMT2A: lysine (K)-specific MethylTransferase 2A
ECM: ExtraCellular Matrix
CXCR4: C-X-C motif chemokine Receptor 4
WHO: World Health Organization
EDTA: EthyleneDiamineTetraacetic Acid
TBS: Tris-buffered saline
DAB: 3,3′-Diaminobenzidine
FFPE: Formalin-Fixed Paraffin Embedded
FAM: 6-carboxyfluorescein
Ct: Cycle threshold
ATCC: American Type Culture Collection
PBS: Phosphate-Buffered Saline
DAPI: 4′,6-diamidino-2-phenylindole
cDNA: complementary DNA
GAPDH: Glycerldehyde-3-Phosphate Deydrogenase
*GUSB*: Glucuronidase Beta
18S: 18S ribosomal RNA
UNG: Uracil N-Glycosylase
SPSS: Statistical Package for Social Science
