# Correction: The nuclear-cytoplasmic trafficking of a chromatin-modifying and remodelling protein (KMT2C), in osteosarcoma

**DOI:** 10.18632/oncotarget.26240

**Published:** 2018-10-12

**Authors:** Caterina Chiappetta, Chiara Puggioni, Raffaella Carletti, Vincenzo Petrozza, Carlo Della Rocca, Claudio Di Cristofano

**Affiliations:** ^1^ UOC of Pathology, Department of Medical-Surgical Sciences and Bio-Technologies, Sapienza University of Rome, Latina, Italy

**This article has been corrected:** The correct author name is given below:

**Claudio Di Cristofano**

Original article: Oncotarget. 2018; 9:30624-30634. https://doi.org/10.18632/oncotarget.25755

